# Draft Genome Sequences of Seven *Streptococcus agalactiae* Strains Isolated from *Camelus dromedarius* at the Horn of Africa

**DOI:** 10.1128/genomeA.00525-17

**Published:** 2017-07-13

**Authors:** Julian Rothen, Tobias Schindler, Joël F. Pothier, Mario Younan, Ulrich Certa, Claudia Daubenberger, Valentin Pflüger, Joerg Jores

**Affiliations:** a Swiss Tropical and Public Health Institute, Basel, Switzerland; b University of Basel, Basel, Switzerland; c Environmental Genomics and Systems Biology Research Group, Institute for Natural Resource Sciences, Zurich University of Applied Sciences (ZHAW), Wädenswil, Switzerland; d Food and Agriculture Organization of the United Nations, Gaziantep, Turkey; e Roche Pharmaceutical Research and Early Development, Department of Pharmaceutical Sciences, Translational Technologies and Bioinformatics, Roche Innovation Center Basel, Basel, Switzerland; f Mabritec AG, Riehen, Switzerland; g International Livestock Research Institute, Nairobi, Kenya; h Vetsuisse-Fakultät Universität Bern, Institut für Veterinär-Bakteriologie, Bern, Switzerland

## Abstract

We present draft whole-genome sequences of seven *Streptococcus agalactiae* strains isolated from *Camelus dromedarius* in Kenya and Somalia. These data are an extension to the group B *Streptococcus* (GBS) pangenome and might provide more insight into the underlying mechanisms of pathogenicity and antibiotic resistance of camel GBS.

## GENOME ANNOUNCEMENT

The natural colonizer of human gastrointestinal and genitourinary tracts *Streptococcus agalactiae*, also known as Lancefield’s group B *Streptococcus* (GBS), is an emerging pathogen of serious clinical concern (1). As a main causative agent of meningitis, sepsis, and respiratory diseases in neonates, GBS is strongly linked to child mortality and morbidity (2). *S. agalactiae* has also been isolated from both healthy and diseased camels in countries from the Horn of Africa (3–7). Given the fundamental role of camels for human nutrition and financial safety in these regions, GBS-associated diseases, such as mastitis or udder abscesses resulting in significant losses in milk production, can have a devastating impact (5). Here, we report the whole-genome sequences of seven GBS strains, isolated from Kenyan and Somalian camels (*Camelus dromedarius*). Previous genomic analysis of these isolates by multilocus sequence typing (MLST) indicated a detached phylogenetic relationship compared to GBS strains of human or bovine origin (5). The three isolates ILRI025, ILRI030, and ILRI067 were isolated from healthy camels, while ILRI037 (causing gingivitis), ILRI054 (causing wound infection), ILRI120 (causing chronic cough), and ILRI127 (causing periarthricular abscess) were associated with disease.

Genomic DNA was extracted from a single bacterial colony cultivated on Columbia sheep blood agar using the QIAamp DSP DNA minikit (Qiagen, Hilden, Germany). DNA was fragmented by ultrasonication using the Covaris S2 instrument (Covaris, Inc., Woburn, MA, USA). Barcoded libraries were generated with the Ion fragment library kit and Ion Xpress DNA barcode adaptors (Life Technologies, Inc., Carlsbad, CA, USA). Sequencing was performed on an Ion Torrent Personal Genome Machine (PGM) system, with the Ion PGM sequencing 400 kit and the Ion 318 Chip version 2 (Life Technologies, Inc.). After sequencing, single processing and base calling were performed using Torrent Suite 3.6 (Life Technologies, Inc.), and barcode-separated FASTQ files were generated. For *de novo* assemblies, we used MIRA version 4.0 (8). Contigs were sorted along the already published (9) GBS genomes of ILRI112 (accession no. HF952106) and ILRI005 (accession no. HF952105) (only for isolate ILRI067) using the Move Contigs function in Mauve version 2.3.1 (10). SeqMan Pro from the Lasergene genomics package version 12.1.0 (DNAStar, Madison, WI) was used to check and manually close gaps between contigs. Genome annotation was added using the NCBI Prokaryotic Genome Annotation Pipeline (PGAP). The seven genomes displayed an overall size between 1,973,342 and 2,049,911 bp, with 1,812 to 1,954 proteins detected (Table 1).

**TABLE 1:** List of *Streptococcus agalactiae* draft whole genomes released to GenBank

Strain	GenBank accession no.	Multilocus ST^a^	Serotype	Genome size (bp)	No. of proteins
ILRI025	NDGG00000000	610	VI	2,013,384	1,876
ILRI030	NDGF00000000	617	VI	1,999,626	1,883
ILRI037	NDGE00000000	612	Ia	2,020,002	1,895
ILRI054	NDGD00000000	615	II	2,021,031	1,867
ILRI067	NDGC00000000	614	V	1,980,469	1,812
ILRI120	NDGB00000000	618	Ia	2,049,911	1,954
ILRI127	NDGA00000000	613	Ia	1,973,342	1,875

^a^ ST, sequence type.

The draft genome sequences of cameloid GBS isolates presented here are a valuable addition to the pangenome of *S. agalactiae* (11). These genomic data provide a basis for the investigation of adaptive factors in GBS host colonization as well as underlying mechanisms of antibiotic resistance development and pathogenicity of camel *S. agalactiae*.

### Accession number(s).

The annotated draft whole-genome sequences of the seven *S. agalactiae* isolates were deposited in GenBank under BioProject no. PRJNA382326. The accession numbers for each isolate are shown in Table 1.

## References

[1] Huber CA, McOdimba F, Pflueger V, Daubenberger CA, Revathi G 2011 Characterization of invasive and colonizing isolates of *Streptococcus agalactiae* in East African adults. J Clin Microbiol 49:3652–3655. doi:10.1128/JCM.01288-11. 21865428 PMC3187314

[2] Schrag SJ, McGee L, Verani J 2010 Prevention of perinatal group B streptococcal disease—revised guidelines from CDC. MMWR Morb Mortal Wkly Rep 59:1–32. 21088663

[3] Edelstein RM, Pegram RG 1974 Contagious skin necrosis of Somali camels associated with *Streptococcus agalactiae*. Trop Anim Health Prod 6:255–256. doi:10.1007/BF02383286. 4617946

[4] Bekele T, Molla B 2001 Mastitis in lactating camels (*Camelus dromedarius*) in Afar Region, north-eastern Ethiopia. Berl Münch Tierarztl Wochenschr 114:169–172. 11413707

[5] Fischer A, Liljander A, Kaspar H, Muriuki C, Fuxelius HH, Bongcam-Rudloff E, de Villiers EP, Huber CA, Frey J, Daubenberger C, Bishop R, Younan M, Jores J 2013 Camel *Streptococcus agalactiae* populations are associated with specific disease complexes and acquired the tetracycline resistance gene *tetM* via a Tn*916*-like element. Vet Res 44:86. doi:10.1186/1297-9716-44-86. 24083845 PMC3850529

[6] Younan M, Bornstein S 2007 Lancefield group B and C streptococci in East African camels (*Camelus dromedarius*). Vet Rec 160:330–335. doi:10.1136/vr.160.10.330. 17351174

[7] Abera M, Abdi O, Abunna F, Megersa B 2010 Udder health problems and major bacterial causes of camel mastitis in Jijiga, Eastern Ethiopia: implication for impacting food security. Trop Anim Health Prod 42:341–347. doi:10.1007/s11250-009-9424-6. 19731066

[8] Chevreux B, Wetter T, Suhai S 1999 Genome sequence assembly using trace signals and additional sequence information, p 45–56. *In* Computer science and biology. Proceedings of the German Conference on Bioinformatics, GCB ’99. GCB, Hannover, Germany.

[9] Zubair S, de Villiers EP, Younan M, Andersson G, Tettelin H, Riley DR, Jores J, Bongcam-Rudloff E, Bishop RP 2013 Genome sequences of two pathogenic *Streptococcus agalactiae* isolates from the one-humped camel *Camelus dromedarius*. Genome Announc 1(4):e00515-13. doi:10.1128/genomeA.00515-13. 23868134 PMC3715676

[10] Darling ACE, Mau B, Blattner FR, Perna NT 2004 Mauve: multiple alignment of conserved genomic sequence with rearrangements. Genome Res 14:1394–1403. doi:10.1101/gr.2289704. 15231754 PMC442156

[11] Tettelin H, Masignani V, Cieslewicz MJ, Donati C, Medini D, Ward NL, Angiuoli SV, Crabtree J, Jones AL, Durkin AS, Deboy RT, Davidsen TM, Mora M, Scarselli M, Margarit y Ros I, Peterson JD, Hauser CR, Sundaram JP, Nelson WC, Madupu R, Brinkac LM, Dodson RJ, Rosovitz MJ, Sullivan SA, Daugherty SC, Haft DH, Selengut J, Gwinn ML, Zhou L, Zafar N, Khouri H, Radune D, Dimitrov G, Watkins K, O’Connor KJ, Smith S, Utterback TR, White O, Rubens CE, Grandi G 2005 Genome analysis of multiple pathogenic isolates of *Streptococcus agalactiae*: implications for the microbial “pan-genome.” Proc Natl Acad Sci U S A 102:13950–13955. doi:10.1073/pnas.0506758102. 16172379 PMC1216834

